# Implant Prosthodontic Rehabilitation after Surgical Treatment for an Oropharyngeal Malignant Tumour Using Tantalum Dental Implants

**DOI:** 10.1155/2021/5585181

**Published:** 2021-04-22

**Authors:** Marko Vuletić, Ivica Pelivan, Dragana Gabrić

**Affiliations:** ^1^Department of Oral Surgery, School of Dental Medicine, University of Zagreb, Zagreb, Croatia; ^2^Department of Prosthodontics, School of Dental Medicine, University of Zagreb, Zagreb, Croatia

## Abstract

Oropharyngeal cancer (OPC) represents a significant portion of head and neck cancers. In most cases, it is localised in the soft palate, lingual and palatine tonsils, base of the tongue, and the surrounding tissues. Alcohol and tobacco exposure are well-known evidence-based risk factors for developing OPC; however, over the last decade, there has been a rapid increase in OPC linked to human papillomavirus (HPV). Dental implant therapy faces many challenges related to immediate and long-term success, and patients who are rehabilitated with implant prosthodontic therapy often have numerous comorbidities. Tantalum is a rare transitional metal element which has high corrosion resistance and is extremely inert. Porous tantalum trabecular metal (PTTM) has high volumetric porosity, a low modulus of elasticity, and very high friction. PTTM implant surface enhancement allows “osseoincorporation,” which means the neovascularisation and formation of new bone directly onto the implant. A 65-year-old patient presented to the Department of Oral Surgery of Clinical Hospital Centre Zagreb after resection of the mandible due to OPC had oral rehabilitation. Three Zimmer Biomet Trabecular Metal™ implants (4.1 × 10 mm) were inserted in the area of lower left first incisor, lower left second premolar, and lower right second premolar, and after four months, a new upper partial denture and the bar-retained mandibular overdenture were made. Implant prosthodontic rehabilitation of head and neck cancer patients is usually challenging in terms of achieving an improvement in its main aim, quality of life; however, today it is a safe and reliable therapy. Although radiation therapy may negatively affect the patient's oral condition and influence the short- and long-term success of the implant, the presented case report showed that the excellent properties of PTTM-enhanced dental implants may give great basis for future comparative researches of using these implants in the treatment of oncologic patients.

## 1. Introduction

Squamous cell carcinoma affecting the head and neck is the sixth most common cancer worldwide, with 700,000 cases diagnosed per year [1]. Despite advances in multimodality therapy, the survival rate remains approximately 40–50% [2, 3]. Oropharyngeal cancer (OPC) represents a significant portion of head and neck cancers [4]. In most cases, it is localised in the soft palate, lingual and palatine tonsils, base of the tongue, and the surrounding tissues. Alcohol and tobacco exposure are well-known evidence-based risk factors for developing OPC; however, over the last decade, there has been a rapid increase in OPC linked to human papillomavirus (HPV) [5]. HPV is a DNA virus with a predilection for the squamous epithelium [6]. The epithelium covering the base of the tongue and tonsils acts as a reservoir for the virus, providing access to the basal layer for viral replication [4]. HPV infection of the oropharynx occurs five times more frequently than infection of the oral cavity, hypopharynx, and larynx because of its predilection for the reticulated crypt epithelium covering the oropharynx, which is unique to the head and neck region [7]. The majority of OPC cases related to HPV are caused by HPV-16. The recommended treatment options for OPC depend on the tumour, mode, metastasis (TNM) stage, the patient's preferences and comorbidities, and the physician's experience. Early-stage disease is often treated with single modality treatment, such as radiotherapy or surgery alone, while regionally advanced OPC is treated with dual modality treatment [8].

Dental implant therapy faces many challenges related to immediate and long-term success, and patients who are rehabilitated with implant prosthodontic therapy often have numerous comorbidities. There have been many efforts to improve dental implant performance at the point of implant-bone contact by modifying the topography and chemistry of the surface [9]. Microtopography of the surface in titanium implants was modified using sandblasting and acid-etch treatment [10]. Many studies published since the 1970s have reported that the size of the pores and percentage of porosity are the most important factors for bone healing around the dental implant [11]. In their 1999 study, Bobyn et al. [12] presented a tantalum-based, trabecular-structured biomaterial with 80% porosity, produced by coating a vitreous carbon scaffold with elemental tantalum through a chemical vapour deposition process (Trabecular Metal Material; Zimmer Biomet TMT, Parsippany, NJ, USA). Tantalum is a rare transitional metal element which has high corrosion resistance and is extremely inert such that it can only be dissolved by hydrofluoric acid and acid solutions containing fluoride and sulphur trioxide. Its inertness is ideal for the fabrication of orthopaedic implants, devices for nerve repair, electrodes for pacemakers, cranioplasty plates, and radiographic markers [13]. The development of porous tantalum metal has resulted in stronger and more biocompatible craniofacial, orthopaedic, and dental implants. Porous tantalum trabecular metal (PTTM) has high volumetric porosity, a low modulus of elasticity, and very high friction [13]. PTTM allows implant surface enhancement, not only for bone ongrowth but also bone ingrowth. This structure allows “osseoincorporation,” which means the neovascularisation and formation of new bone directly onto the implant [14]. For dental applications, this PTTM biomaterial is positioned in the midsection of a conventional titanium alloy implant body (Trabecular Metal™ Dental Implants; Zimmer Biomet, Palm Beach Gardens, FL, USA), comprising the coronal, apical, and internal regions of the implant [15].

This case report presents the successful clinical use of TM dental implants in an irradiated mandible for the oral rehabilitation of an oncologic patient.

## 2. Case Presentation

**Figure 1:**
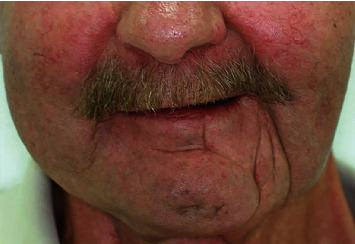
Frontal view of the patient.

**Figure 2:**
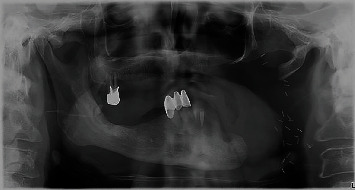
Panoramic image.

A 65-year-old male patient presented to the Department of Oral Surgery of Clinical Hospital Centre Zagreb after resection of the mandible due to OPC that was surgically treated in April 2015 (Figures 1 and 2). He was referred by a prosthodontist due to an inability to function with a full denture. He had no history of tobacco smoking and only drank socially and did not have any other usual predisposition factors for carcinogenesis in this area. Until this disease, his medical status was remarkable. The patient's medical history revealed radical neck dissection, commando surgery, and reconstruction using an anterolateral thigh (ALT) flap due to a squamous cell carcinoma localised in the anterior palate arch with expansion into the retromolar trigonum area and lateral oropharyngeal walls (T3N0M0). The patient had received postoperative chemotherapy (cisplatin i.v.) for a total of three cycles in conjunction with radiotherapy. Three-dimensional conformal radiotherapy was performed in the region of the tumour, with a total of 32 fractions using a total dose of 64 Gy. In the region of the neck lymph node bilaterally 25 fractions in a total dose of 50 Gy were performed. The patient handled the therapy well (ECOG 0), with no major side effects. The only symptom he had was the expected local side effect of mucositis. Replacement hormone therapy with 50 *μ*g levothyroxine had also been introduced.

**Figure 3:**
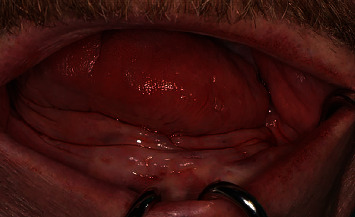
Intraoral situation before surgery.

On initial examination, an edentulous mandible was found with a narrow zone of attached mucosa, preventing the fabrication of a satisfactory full denture (Figure 3). Three months before, the patient extracted all remained teeth, upper right molar, four mandibular incisors, and lower right canine because of impossibility of restoration due to deep caries or periapical lesions. A cone beam computed tomography (CBCT) was performed, where adequate residual alveolar ridge height and width for the insertion of dental implants were observed. After consultation with a prosthodontist, we decided to place three dental implants for future fabrication of a bar-retained overdenture.

**Figure 4:**
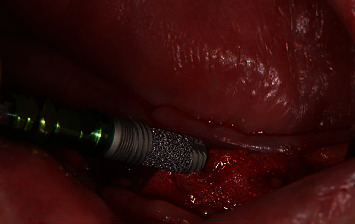
Insertion of Trabecular Metal™ Dental Implants (Zimmer Biomet, Palm Beach Gardens, FL, USA).

**Figure 5:**
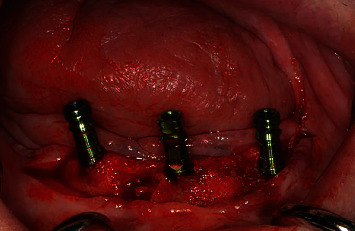
Clinical view after insertion.

**Figure 6:**
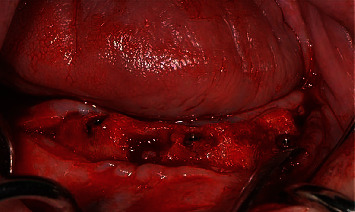
Situation after removing transfers.

**Figure 7:**
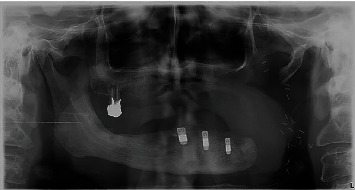
Postoperative panoramic image.

**Figure 8:**
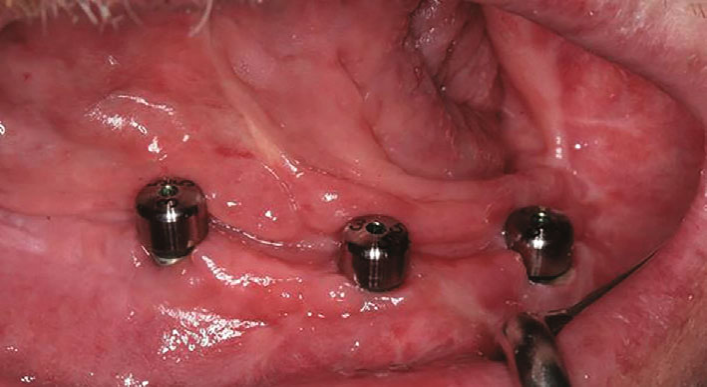
Placing gingival healing abutments.

**Figure 9:**
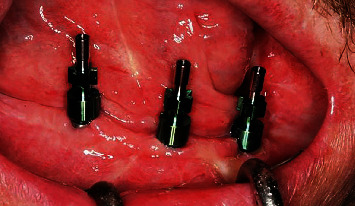
Transfers for open-tray impression.

**Figure 10:**
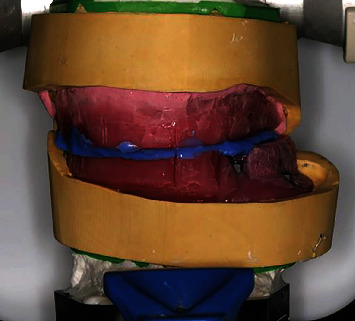
Maxillomandibular relationship.

**Figure 11:**
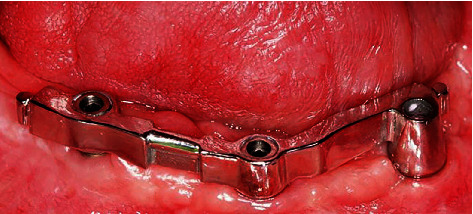
Individual milled bar for retaining mandibular overdenture.

**Figure 12:**
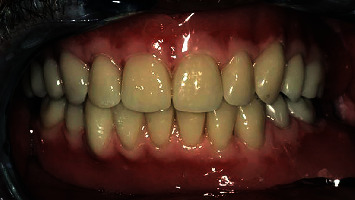
Upper partial denture and the bar-retained mandibular overdenture.

In accordance with the ethical protocol of the School of Dental Medicine of the University of Zagreb, written consent was obtained from the patient prior to surgery in January 2019. The patient received antibiotic premedication (one day before surgery, the patient started antibiotic therapy with amoxicillin with clavulanic acid twice a day and lasted for six days postoperative). Crestal incision on the edentulous mandible was performed under local infiltration anaesthesia (4% articaine with epinephrine 1 : 200,000; 5.4 mL). The full mucoperiosteal flap was elevated to expose the residual alveolar ridge. Three Zimmer Biomet Trabecular Metal™ implants (4.1 × 10 mm) were inserted in the area of lower left first incisor, lower left second premolar, and lower right second premolar with a maximum insertion torque of 25 Ncm (Figures 4–6). Sutures were removed 7 days postoperatively without any complications. Four months after implant placement (Figure 7), the implants were reopened and gingival formers were placed (Figure 8). Two weeks later, anatomical and functional impressions of both jaws were taken for the production of a new upper partial denture and the bar-retained mandibular overdenture (Figures 9–11). The greatest challenge was to reconcile the severely displaced maxilla mandibular relationship with an aesthetic and functional final result. Three weeks later, new dentures were given to the patient (Figure 12). No local complications of implant prosthodontic therapy were clinically observed. The patient reported a significant improvement in chewing efficiency and, consequently, quality of life in period afterwards. The patient was followed up until now for 1 year, through regular examinations every 6 months, and he did not report any problems or we did not notice any potential complications in this period.

## 3. Discussion

Oral rehabilitation after head and neck cancer treatment is one of the most challenging procedures in dentistry today. Patients treated with chemotherapy and radiation are predisposed to hyposalivation, mucositis, reduced angiogenesis, delayed healing, loss of taste, radiation-induced caries, trismus, and osteoradionecrosis of the jaw (ORN). ORN, which is the ischemic necrosis of bone, is the most serious complication [16]. Irradiation causes changes in the bone as a result of injury to the remodelling system (osteocytes, osteoblasts, and osteoclasts) and vascular injury, such as hyperaemia, thrombosis, endarteritis, and a progressive occlusion and obliteration of small vessels. Acellularity and avascularity with fibrosis and fatty degeneration affect the bone marrow in the postradiation period [17]. ORN occurs more frequently in the mandible than the maxilla. In 30% of cases, ORN is asymptomatic, but the remaining cases always develop fistula, pain, or bone fracture. In the literature [18, 19], the risk of developing ORN has been reported to be about 2%, but this depends on the type and invasiveness of the surgical procedure and can be much higher. The risk of trauma-induced ORN is indefinite and is related not only to the volume and radiation dose but also to the dental health of the patient [16, 18]. Because the potential risk persists throughout the patient's life, the placement of dental implants should be carefully planned. The patient in this case report only had mucositis as a side effect of head and neck treatment and showed no signs of ORN after dental extractions, which were performed three months before implant therapy.

**Figure 13:**
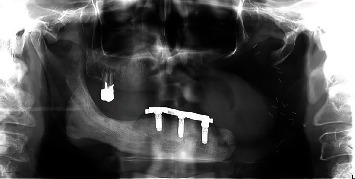
Panoramic image after 1-year follow-up.

An extensive prosthetic treatment is needed to restore function, speech, and mastication. Implant-supported prosthodontics is a challenge, but it has many advantages over conventional prostheses including improved mastication, retention, and patient acceptance [20]. Patients receiving head and neck radiation are at greater risk for dental implant failure due to the changes in hard and soft tissue mentioned earlier. Irreversible changes to the blood vessels and bone-forming cells that affect bone turnover caused by radiation therapy compromise the osseointegration of dental implants [21]. In the literature, there are no clear guidelines regarding the timing of implant placement in patients who have undergone radiotherapy of the jaw. Some authors [22] state that the bone should be allowed to recover from the radiation insult for some time before dental implant therapy, while Schepers et al. [23] reported that radiation therapy after dental implant placement did not affect osseointegration. A review by Nooh [24] found that postimplantation radiation therapy had a slightly better overall dental implant survival rate (92.2%) than preimplantation radiation therapy (88.9%), but these findings did not reach statistical significance. Although the mandible is more prone to ORN, it was reported that dental implants osseointegrate with greater success in the irradiated mandible in regard to its anatomy [25]. The anterior part of the mandible is usually exposed to a lower radiation dose and has a better remodelling capability due to the additional vascular supply from the facial artery [26]. In the presented case report, three dental implants were inserted into the anterior region of the mandible during the postimplantation period, and there were no signs of complications after 1-year follow-up (Figure 13).

Colella et al. [25] reported a correlation between implant failure and a radiation dose greater than 45 Gy. In the review by Nooh [24], radiation doses among individual studies ranged from 25 to 72 Gy; the uncommon incidence of low-dose radiation therapy was also 45 Gy, while a radiation dose above 55 Gy was associated with a significantly reduced survival rate of implants inserted during the postradiation period. The total dose of radiotherapy in the presented patient was 64 Gy because this is the dose prescribed for the treatment of squamous cell carcinoma of the head and neck, which is above marginal value range in which dental implant rehabilitation is recommended.

The recovery period after ionising radiation has a bipolar flow, consisting of a short positive phase lasting a few weeks and a long negative phase that lasts for years, characterised by hypovascularity, hypoxia, and hypocellularity [27]. In the study by Linsen et al. [28], the time between radiation therapy and dental implant placement varied from 6 weeks to 24 months, while other studies [24] reported intervals ranging from 4 to 228 months for the preimplantation period. In most studies, implant therapy was accomplished within a mean of 24 months after irradiation. Although the review by Nooh [24] found no statistical significance between time interval and dental implant survival rate, there was a higher overall implant survival rate after a mean of 30 months following the end of radiation. The recovery period in this case was 40 months after radiation therapy.

In the literature, there are no recent cases or studies involving TM implants in irradiated jaws. Joglekar et al. [29] found that tantalum trabecular metal acetabular components provided reliable reconstructions in patients following prior pelvic radiation. After a 5-year follow-up, they did not report any clinical or radiographic failures. In a study by Bencharit et al. [30], the authors mentioned that modern root-form endosseous titanium dental implants are commonly used due to their high success rate, but it is obvious that its osseointegration should be enhanced in patients with certain systemic medical conditions. In cases with poor tissue healing, such as patients with osteoporosis, diabetes mellitus, or irradiated bone, the findings related to TM implants suggest that there should be benefits from this type of implant. This case report demonstrated oral rehabilitation with TM dental implants with excellent ingrowth promotion for secondary implant stability, although further evidence is needed. During placement, implants were submerged 1 mm below the crestal bone, so from a theoretical point of view, there was no influence of the oral cavity or to bacteria. Spinato et al. [31] presented a case report of a healthy patient with a trabecular metal implant, in whom bone resorption was observed only in the coronal third of the implant, which was primarily composed of titanium. The greatest amount of bone was around the tantalum trabeculae compared to the apical part of the implant. The main reason for this result was bone remodelling following surgery and bone loss due to implant unloading. In many cases, bone disappearance from the coronal third can be related to bone remodelling after surgery and consequent erosion of the basal bone. The amount of bone around the middle trabecular part was greater because of the well-known properties of TM, in addition to the fact that the implant was unloaded. Spinato et al. [31] observed that the architecture of TM not only enhanced bone growth into the pores but also permitted more mechanical retention. Analysis of the orthopantomograph of our patient after 1-year follow-up showed consistent findings to those observed by Spinato et al. [31], who observed a small amount of bone loss around the implant in the coronal part, but without any clinical signs of peri-inflammation.

## 4. Conclusion

Implant prosthodontic rehabilitation of head and neck cancer patients is usually challenging in terms of achieving an improvement in its main aim, quality of life; however, today it is a safe and reliable therapy. Although radiation therapy may negatively affect the patient's oral condition and influence the short- and long-term success of the implant, the presented case report showed that the excellent properties of PTTM-enhanced dental implants may give great basis for future comparative researches of using these implants in the treatment of oncologic patients.

## References

[1] Ferlay J., Soerjomataram I., Dikshit R. (2015). Cancer incidence and mortality worldwide: sources, methods and major patterns in GLOBOCAN 2012. *International Journal of Cancer*.

[2] Allen C., Duffy S., Teknos T. (2007). Nuclear factor-kappaB-related serum factors as longitudinal biomarkers of response and survival in advanced oropharyngeal carcinoma. *Clinical Cancer Research*.

[3] Leibowitz M. S., Andrade Filho P. A., Ferrone S., Ferris R. L. (2011). Deficiency of activated STAT1 in head and neck cancer cells mediates TAP1-dependent escape from cytotoxic T lymphocytes. *Cancer Immunology, Immunotherapy*.

[4] McLaughlin-Drubin M. E., Munger K. (2009). Oncogenic activities of human papillomaviruses. *Virus Research*.

[5] Ducatman B. S. (2018). The role of human papillomavirus in oropharyngeal squamous cell carcinoma. *Archives of Pathology & Laboratory Medicine*.

[6] Munoz N., Castellsagué X., de González A. B., Gissmann L. (2006). Chapter 1: HPV in the etiology of human cancer. *Vaccine*.

[7] Combes J. D., Franceschi S. (2014). Role of human papillomavirus in non-oropharyngeal head and neck cancers. *Oral Oncology*.

[8] Shah J. P., Patel S. G., Singh B. (2012). *Jatin Shah’s Head and Neck Surgery and Oncology*.

[9] Lavenus S., Louarn G., Layrolle P. (2010). Nanotechnology and dental implants. *International Journal of Biomaterials*.

[10] Trisi P., Marcato C., Todisco M. (2003). Bone-to-implant apposition with machined and MTX microtextured implant surfaces in human sinus grafts. *The International Journal of Periodontics & Restorative Dentistry*.

[11] Schlee M., Pradies G., Mehmke W. U. (2015). Prospective, multicenter evaluation of trabecular metal-enhanced titanium dental implants placed in routine dental practices: 1-year interim report from the development period (2010 to 2011). *Clinical Implant Dentistry and Related Research*.

[12] Bobyn J. D., Stackpool G. J., Hacking S. A., Tanzer M., Krygier J. J. (1999). Characteristics of bone ingrowth and interface mechanics of a new porous tantalum biomaterial. *Journal of Bone and Joint Surgery. British Volume (London)*.

[13] Levine B. R., Sporer S., Poggie R. A., Della Valle C. J., Jacobs J. J. (2006). Experimental and clinical performance of porous tantalum in orthopedic surgery. *Biomaterials*.

[14] Cohen R. (2002). A porous tantalum trabecular metal: basic science. *The American Journal of Orthopedics*.

[15] Kim D. J., Huja S. S., Tee B. C. (2013). Bone ingrowth and initial stability of titanium and porous tantalum dental implants: a pilot canine study. *Implant Dentistry*.

[16] Vissink A., Burlage F. R., Spijkervet F. K., Jansma J., Coppes R. P. (2003). Prevention and treatment of the consequences of head and neck radiotherapy. *Critical Reviews in Oral Biology and Medicine*.

[17] Tanaka T. I., Chan H. L., Tindle D. I., Maceachern M., Oh T. J. (2013). Updated clinical considerations for dental implant therapy in irradiated head and neck cancer patients. *Journal of Prosthodontics*.

[18] Nabil S., Samman N. (2012). Risk factors for osteoradionecrosis after head and neck radiation: a systematic review. *Oral Surgery, Oral Medicine, Oral Pathology, Oral Radiology*.

[19] Nabil S., Samman N. (2011). Incidence and prevention of osteoradionecrosis after dental extraction in irradiated patients: a systematic review. *International Journal of Oral and Maxillofacial Surgery*.

[20] Anderson L., Meraw S., Al-Hezaimi K., Wang H. L. (2013). The influence of radiation therapy on dental implantology. *Implant Dentistry*.

[21] Ihde S., Gundlach K., Konstantinovic V. S. (2009). Effects of radiation therapy on craniofacial and dental implants: a review of the literature. *Oral Surgery, Oral Medicine, Oral Pathology, Oral Radiology, and Endodontics*.

[22] Granström G. (2005). Osseointegration in irradiated cancer patients: an analysis with respect to implant failures. *Journal of Oral and Maxillofacial Surgery*.

[23] Schepers R. H., Slagter A. P., Kaanders J. H., van den Hoogen F. J., Merkx M. A. (2006). Effect of postoperative radiotherapy on the functional result of implants placed during ablative surgery for oral cancer. *International Journal of Oral and Maxillofacial Surgery*.

[24] Nooh N. (2013). Dental implant survival in irradiated oral cancer patients: a systematic review of the literature. *The International Journal of Oral & Maxillofacial Implants*.

[25] Colella G., Cannavale R., Pentenero M., Gandolfo S. (2007). Oral implants in radiated patients: a systematic review. *The International Journal of Oral & Maxillofacial Implants*.

[26] de Oliveira J. A., do Amaral Escada A. L., Alves Rezende M. C., Mathor M. B., Alves Claro A. P. R. (2012). Analysis of the effects of irradiation in osseointegrated dental implants. *Clinical Oral Implants Research*.

[27] Visch L. L., van Waas M. A., Schmitz P. I., Levendag P. C. (2002). A clinical evaluation of implants in irradiated oral cancer patients. *Journal of Dental Research*.

[28] Linsen S. S., Martini M., Stark H. (2012). Long-term results of endosteal implants following radical oral cancer surgery with and without adjuvant radiation therapy. *Clinical Implant Dentistry and Related Research*.

[29] Joglekar S. B., Rose P. S., Lewallen D. G., Sim F. H. (2012). Tantalum acetabular cups provide secure fixation in THA after pelvic irradiation at minimum 5-year followup. *Clinical Orthopaedics and Related Research*.

[30] Bencharit S., Byrd W. C., Altarawneh S. (2014). Development and applications of porous tantalum trabecular metal-enhanced titanium dental implants. *Clinical Implant Dentistry and Related Research*.

[31] Spinato S., Zaffe D., Felice P., Checchi L., Wang H. L. (2014). A trabecular metal implant 4 months after placement: clinical-histologic case report. *Implant Dentistry*.

